# Structural brain correlates of non‐verbal cognitive ability in 5‐year‐old children: Findings from the FinnBrain birth cohort study

**DOI:** 10.1002/hbm.26463

**Published:** 2023-08-22

**Authors:** Elmo P. Pulli, Saara Nolvi, Eeva Eskola, Elisabeth Nordenswan, Eeva Holmberg, Anni Copeland, Venla Kumpulainen, Eero Silver, Harri Merisaari, Jani Saunavaara, Riitta Parkkola, Tuire Lähdesmäki, Ekaterina Saukko, Eeva‐Leena Kataja, Riikka Korja, Linnea Karlsson, Hasse Karlsson, Jetro J. Tuulari

**Affiliations:** ^1^ FinnBrain Birth Cohort Study, Turku Brain and Mind Center, Department of Clinical Medicine University of Turku Turku Finland; ^2^ Centre for Population Health Research Turku University Hospital and University of Turku Turku Finland; ^3^ Turku Institute for Advanced Studies, Department of Psychology and Speech‐Language Pathology University of Turku Turku Finland; ^4^ Department of Psychology University of Turku Turku Finland; ^5^ Department of Radiology University of Turku Turku Finland; ^6^ Department of Medical Physics Turku University Hospital and University of Turku Turku Finland; ^7^ Department of Radiology Turku University Hospital Turku Finland; ^8^ Pediatric Neurology, Department of Pediatrics and Adolescent Medicine Turku University Hospital and University of Turku Turku Finland; ^9^ Department of Pediatrics and Adolescent Medicine Turku University Hospital and University of Turku Turku Finland; ^10^ Department of Psychiatry Turku University Hospital and University of Turku Turku Finland; ^11^ Turku Collegium for Science, Medicine and Technology University of Turku Turku Finland; ^12^ Department of Psychiatry University of Oxford Oxford UK

**Keywords:** cortex, intelligence, MRI, neuroimaging, Qdec

## Abstract

Non‐verbal cognitive ability predicts multiple important life outcomes, for example, school and job performance. It has been associated with parieto–frontal cortical anatomy in prior studies in adult and adolescent populations, while young children have received relatively little attention. We explored the associations between cortical anatomy and non‐verbal cognitive ability in 165 5‐year‐old participants (mean scan age 5.40 years, SD 0.13; 90 males) from the FinnBrain Birth Cohort study. T1‐weighted brain magnetic resonance images were processed using FreeSurfer. Non‐verbal cognitive ability was measured using the Performance Intelligence Quotient (PIQ) estimated from the Block Design and Matrix Reasoning subtests from the Wechsler Preschool and Primary Scale of Intelligence (WPPSI‐III). In vertex‐wise general linear models, PIQ scores associated positively with volumes in the left caudal middle frontal and right pericalcarine regions, as well as surface area in left the caudal middle frontal, left inferior temporal, and right lingual regions. There were no associations between PIQ and cortical thickness. To the best of our knowledge, this is the first study to examine structural correlates of non‐verbal cognitive ability in a large sample of typically developing 5‐year‐olds. The findings are generally in line with prior findings from older age groups, with the important addition of the positive association between volume / surface area in the right medial occipital region and non‐verbal cognitive ability. This finding adds to the literature by discovering a new brain region that should be considered in future studies exploring the role of cortical structure for cognitive development in young children.

## Graphical abstract

The structural cortical correlates of non‐verbal ability in typically developing 5‐year‐olds were for the most part similar to those seen in older participants in earlier studies. The novel finding in our study was the positive association between volume/surface area in the right medial occipital region and non‐verbal ability.

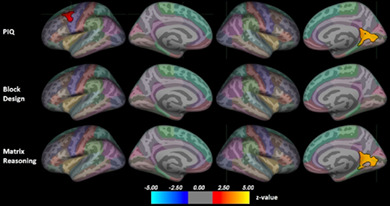

## 1 INTRODUCTION

Cognitive ability is an important predictor for many important life outcomes (Plomin & Von Stumm, 2018), such as school and academic performance (Deary et al., 2007; Neisser et al., 1996; Strenze, 2007), educational attainment (M. I. Brown et al., 2021), occupational status (M. I. Brown et al., 2021; Lang & Kell, 2020; Schmidt & Hunter, 2004; Strenze, 2007), job performance (Bertua et al., 2005; Neisser et al., 1996; Schmidt & Hunter, 2004, 1998; N. Schmitt, 2014), income (M. I. Brown et al., 2021; Furnham & Cheng, 2017; Lang & Kell, 2020; Neisser et al., 1996), life expectancy (Batty et al., 2007; Whalley & Deary, 2001), and other psychiatric and somatic health outcomes (e.g., alcohol use, see Batty et al., 2006; and obesity, see Chandola et al., 2006). Cognitive ability is considered stable (Deary et al., 2013; Gow et al., 2011) and highly genetically determined (Deary et al., 2006; Plomin & Von Stumm, 2018) individual characteristic in adult populations, while environmental factors play a greater role the younger the subjects are (Haworth et al., 2009; Plomin et al., 1997; Plomin & Von Stumm, 2018). General cognitive ability can be divided into verbal and non‐verbal ability. Based on current evidence in school‐age children and adolescents, verbal ability is associated with structural and functional neural features in language areas (Khundrakpam et al., 2017; Qi et al., 2019; Ramsden et al., 2011), while non‐verbal ability is associated with structural and functional features in (pre)motor areas (Kim et al., 2016; Ramsden et al., 2011). Furthermore, cognitive ability and brain structural features (volume, surface area, and cortical thickness) are highly heritable in both children (Deary et al., 2006; Jha, Xia, Schmitt, et al., 2018; Lenroot et al., 2009; J. E. Schmitt, Raznahan, et al., 2019; Wallace et al., 2006) and adults (Deary et al., 2006; Panizzon et al., 2009; Posthuma et al., 2002; J. E. Schmitt, Raznahan, et al., 2019; Thompson et al., 2001; Winkler et al., 2010).

The developmental research on brain–cognitive correlates is challenged by the dynamic development of brain across the increasing age. The brain grows rapidly in the first years of life, reaching approximately 80% of adult volume by the age 2 years (Knickmeyer et al., 2008), and 95% by the age 6 years (Phan et al., 2018). Total gray matter (GM) volume reaches its peak at approximately 6 years of age (Bethlehem et al., 2022; Courchesne et al., 2000). The development of GM volumes varies depending on the region, wherein frontal and temporal regions show peak volumes in late childhood, while parietal and occipital volumes are already decreasing by the age 5 years (Aubert‐Broche et al., 2013; Bethlehem et al., 2022). In turn, cortical surface area shows global increase in early childhood and reaches its peak at approximately 10–12 years of age (Bethlehem et al., 2022; T. T. Brown et al., 2012; Raznahan et al., 2011; Wierenga et al., 2014). There has been controversy regarding the developmental trajectory of cortical thickness with estimates of the age of peak cortical thickness varying from early to late childhood (Walhovd et al., 2016). However, a recent study combining data from over 100 studies and 100,000 scans has concluded that cortical thickness peaks as early as the second year of life (Bethlehem et al., 2022). Notably, some earlier studies have found different developmental trajectories of cortical thickness development depending on the child's cognitive ability (Khundrakpam et al., 2017; Shaw et al., 2006), challenging the idea that simply being further on the typical developmental neural trajectory would correlate with higher cognitive ability. As such, it is important to explore longitudinal samples to characterize the potential individual differences in the developmental trajectories. However, in many previous longitudinal studies on the topic, the follow‐up only starts at approximately 5 years of age (Khundrakpam et al., 2017; Shaw et al., 2006; Sowell et al., 2004), losing statistical power in the youngest age groups. Therefore, large cross‐sectional samples can be especially useful to provide new information in the less explored young age groups.

The Parieto–Frontal Integration Theory (P–FIT) model proposes that cognitive ability is consistently associated with structural and functional features of a network including widespread frontal and parietal regions, the anterior cingulate cortex, and sensory regions within the temporal and occipital lobes (based on a review of the literature, see Jung & Haier, 2007). A more recent meta‐analysis of structural and functional neuroimaging studies found generally good agreement with the P–FIT model, however there was discrepancy in the results regarding the temporal and occipital regions, for example, related to task vs. resting state functional imaging (Basten et al., 2015). However, these findings are mostly based on adult studies (Basten et al., 2015 excluded studies in children and adolescents from their review), while the neural bases of cognitive ability at different ages and developmental stages throughout childhood are not as well understood.

In line with the P‐FIT model, previous studies on school‐age children and adolescents have found positive associations between general cognitive ability and GM volume in frontal (Pangelinan et al., 2011; Reiss et al., 1996) and parietal lobes (Pangelinan et al., 2011). One study found prefrontal cortical GM volume to predict approximately 20% of the variance in cognitive ability (greater volume predicted higher cognitive ability) in children between the ages 5 and 17 years (Reiss et al., 1996). Additionally, studies of children and adolescents have found negative associations between general cognitive ability and the volumes in the right middle temporal gyrus (Yokota et al., 2015, participants separated into clusters with different profiles of cognitive ability) as well as positive associations between general cognitive ability and GM volumes in the whole brain and the bilateral cingulate gyrus (Wilke et al., 2003, effects were driven by the adolescents). There is some evidence that surface area is also positively associated with general cognitive ability from birth to 11 years of age (Girault et al., 2020; Schnack et al., 2015; Sølsnes et al., 2015) and that children with higher cognitive ability reach the maximal surface area faster (Schnack et al., 2015). Furthermore, greater prefrontal surface area has been linked to higher general cognitive ability in children aged 9–11 years (Vargas et al., 2020). However, pediatric studies examining the connection between surface area and cognitive ability are scarce relative to studies using cortical thickness as a brain measure of interest.

Similarly in line with the P‐FIT model, thicker cortex in frontal and parietal regions may predict later higher verbal ability in infants (Girault et al., 2020) or academic achievement in adolescents (Meruelo et al., 2019). Similarly, studies have found positive associations between non‐verbal ability and cortical thickness in frontal regions in 4–7‐year‐old children (with low socioeconomic status, please see Leonard et al., 2019) and adolescents (Schilling et al., 2013). On the other hand, a study of 12–14‐year‐olds found negative associations between general cognitive ability and cortical thickness in bilateral parietal regions (Squeglia et al., 2013). Similarly, one study found negative associations between cortical thickness and working memory in 4–8‐year‐olds in superior and middle frontal, superior parietal, and anterior cingulate regions (Botdorf & Riggins, 2018), while another found no correlations between cortical thickness and working memory in any brain regions in 6–16‐year‐olds (Faridi et al., 2015). Furthermore, a recent longitudinal study in children and adolescents found positive correlations between general cognitive ability and cortical thickness mostly in the superior frontoparietal cortex, frontopolar cortex, and language centers (J. E. Schmitt, Raznahan, et al., 2019), which are among the regions typically associated with cognitive ability according to the P‐FIT model (Jung & Haier, 2007). Notably, correlations were modest in young children but became stronger at approximately 10 years of age (J. E. Schmitt, Raznahan, et al., 2019). Some other studies have also focused on this dynamic development of cortical thickness in childhood and adolescence: One study found greater vocabulary improvement associated with greater thinning between the ages 5 and 11 years in widespread brain regions especially in the left hemisphere (Sowell et al., 2004). In another study, the correlation between general cognitive ability and cortical thickness was negative until about 8 years of age and then turned positive (Shaw et al., 2006).

In summary, most studies examining brain structure and cognitive ability are conducted in samples with wide age ranges typically focusing on late childhood and adolescence, while such research in younger age groups is scarcer. Notably, studies with wider age ranges risk conflating findings from different age groups, and studies with large samples from a limited age range are warranted to better explore the neural basis of cognitive ability at the specific developmental stage. To the best of our knowledge, there are no previous large neuroimaging studies focusing solely on typically developing 5‐year‐olds. Five years is a particularly interesting age to study the structural brain correlates of cognitive ability, as the children are old enough to both cooperate in cognitive assessment to be reliably evaluated and to lie still in the scanner while awake. Furthermore, 5‐year‐olds have yet to start school (in Finland), meaning most of them have not gone through the changes associated with the learning of academic abilities such as reading (Chyl et al., 2021) and arithmetic (Hashimoto et al., 2022).

In the current study, we examined cortical structural correlates of non‐verbal ability at 5 years of age. More specifically, we explored the associations between cortical gray matter volume, surface area, and cortical thickness and non‐verbal ability measured with Block Design and Matrix Reasoning tasks from the Wechsler Preschool and Primary Scale of Intelligence (WPPSI‐III; Wechsler, 1967) in a sample of 165 typically developing 5‐year‐olds participating in a larger birth cohort follow‐up. Based on previous research, we expected non‐verbal ability to be positively associated with volume and surface area in frontal and parietal regions. We also expected to find associations between cognitive ability and cortical thickness in frontal and parietal regions, but we did not set an explicit hypothesis for the direction of the association, as the findings from previous cortical thickness studies are conflicting and studies regarding a similar age group are rare. Finally, due to the scarcity of previous research on this topic in this age group, this study is also exploratory in the sense that we conduct vertex‐wise analyses for the whole cortex, and we performed all analyses so that they test both positive and negative associations.

## 2 METHODS

This study was conducted in accordance with the Declaration of Helsinki, and it was approved by the Joint Ethics Committee of the University of Turku and the Hospital District of Southwest Finland: (1) ETMK: 26/1801/2015 for the neuropsychological measurements, and (2) ETMK: 31/180/2011 for the neuroimaging.

### 2.1 Participants

The participants are a part of the FinnBrain Birth Cohort Study (www.finnbrain.fi), which prospectively examines the influence of genetic and environmental factors on child development and later health outcomes (Karlsson et al., 2018). Pregnant women (*n* = 3808) attending their first trimester ultrasound at gestational week (GW) 12, their spouses (*n* = 2623), and babies to‐be born (*n* = 3837; including 29 twin pairs) were recruited in Southwest Finland between December 2011 and April 2015. Ultrasound‐verified pregnancy and a sufficient knowledge of Finnish or Swedish languages were required for participation. The cohort study includes several follow‐up studies. Those participants that attended neuropsychological and neuroimaging visits as part of the 5‐year data collection were included in this study.

The participants were first recruited to the neuropsychological assessments at 5 years of age. The participants recruited for this visit were focus cohort families (highest or lowest quartile scores of maternal prenatal distress, please see Karlsson et al., 2018 for more details) and families who had actively participated in previous FinnBrain study visits. For the neuroimaging visit, we primarily recruited participants that had attended the neuropsychological visit. For the neuropsychological visits, 1288 families were contacted and informed of the study, and of these families 974 (75.6%) were reached by telephone. From all the contacted families, 545 (42.3%) participated in a study visit (304 boys (55.8%), mean age 5.01 (SD 0.08), range 4.89–5.37 years). For the neuroimaging visits, 541 families were contacted and 478 (88.4%) of them were reached. In total, 203 (37.5%) participants attended imaging visits (113 boys (55.7%), mean age 5.40 (SD 0.13), range 5.08–5.79 years). Altogether 196 participants attended both visits.

We originally aimed to scan all subjects between the ages 5 years 3 months and 5 years 5 months. The age range was selected partially due to overall collection schedule, as the FinnBrain study had other ongoing visits at approximately 5 years of age that were conducted in a certain order. Simultaneously, we wanted the sample to accurately represent the developmental stage at a specific age and opted for a 2‐month range for the target age for neuroimaging. However, there was a pause in visits due to the start of the COVID‐19 pandemic, and subsequently many of the participants were older than planned when they were scanned (152/203 (74.9%) of the participants attended the visit within the intended age range). The exclusion criteria for the neuroimaging study were: (1) born before GW 35 (before GW 32 for those with exposure to maternal prenatal synthetic glucocorticoid treatment), (2) developmental anomaly or abnormalities in senses or communication (e.g., blindness, deafness, congenital heart disease), (3) known long‐term medical diagnosis (e.g., epilepsy, autism), (4) ongoing medical examinations or clinical follow up in a hospital (meaning there has been a referral from primary care setting to special health care), (5) child use of continuous, daily medication (including per oral medications, topical creams and inhalants; One exception to this was desmopressin medication, which was allowed), (6) history of head trauma (defined as concussion necessitating clinical follow up in a health care setting or worse), (7) metallic (golden) ear tubes (to assure good‐quality scans), and routine magnetic resonance imaging (MRI) contraindications.

For this study, only participants with an adequate quality T1 image (*n* = 173/203, assessed by Elmo P. Pulli as described in Pulli et al., 2022) and successful assessment of cognition (*n* = 166/173) were included. Additionally, one participant was excluded due to scoring below 4 scaled score in verbal ability test Similarities and below the standard score 70 assessed by the performance intelligence quotient (PIQ; calculated from Block Design and Matrix Reasoning scaled scores and the estimated scaled score for a third non‐verbal subtest, see more detailed description later in the Methods), leaving us with a final sample size of 165 participants. Of this sample, 115 (70%) were scanned before the start of the COVID‐19 pandemic. After a small break, the visit continued until March 2021. A few participants were missing one of the non‐verbal tasks, and missing data were not imputed. Consequently, the sample sizes were 164 for the Matrix Reasoning task, 160 for the Block Design task, and 159 for PIQ. None of the participants had started in formal education (primary school) as per the Finnish school system. The characteristics of the final sample (*n* = 165) are displayed in Table 1.

**TABLE 1:** Participant demographics and maternal medical history variables.

Continuous variables	Mean	SD	Min	Max
Age at scan (years)	5.40	0.13	5.08	5.79
Age at cognitive assessment (years)	5.01	0.08	4.92	5.31
Ponderal index	14.07	1.19	11.21	17.63
Gestational age at birth (weeks)	39.79	1.57	33.86	42.29
Birth weight (grams)	3566	471	2450	4980
Maternal age at term (years)	31.0	4.6	19.1	41.3
Maternal BMI before pregnancy	24.2	4.3	17.5	42.0
5 min Apgar score	9.12	0.66	4	10
Distress score, GW 14	7.63	7.08	0	39
Distress score, GW 24	7.95	7.12	0	34
Distress score, GW 34	7.67	7.51	0	39
Prenatal distress sum score	23.26	19.14	0	96
Distress score, month 3	6.64	6.44	0	30
Distress score, month 6	7.64	7.36	0	43
Postnatal distress sum score	14.28	12.39	0	70

Categorical variables	Number	Percent
*Sex*		
Male	91	55.2
Female	74	44.8
*Maternal education level*		
Low	34	20.6
Middle	46	27.9
High	85	51.5
*Paternal education level*		
Low	29	17.6
Middle	39	23.6
High	40	24.2
Missing	57	34.5
*Maternal monthly income, estimated after taxes (euros)*		
≤ 1500	53	32.1
1501–2500	88	53.3
2501–3500	15	9.1
≥ 3501	3	1.8
Missing	6	3.6
*Maternal background*		
Finnish	156	94.5
Other	4	2.4
Missing	5	3
*Gestational age at birth*		
Term, gestational weeks ≥ 37	156	94.5
Preterm, gestational weeks < 37	9	5.5
*Stay in neonatal intensive care unit*		
Yes	23	13.9
No	142	86.1
*Alcohol use during pregnancy*		
Yes, continued to some degree after learning about pregnancy	15	9.1
Yes, stopped after learning about pregnancy	30	18.2
No	112	67.9
Missing	8	4.8
*Tobacco smoking during pregnancy*		
Yes	11	6.7
No	154	93.3
*Illicit drug use during pregnancy*		
No	157	95.2
Missing	8	4.8
*Pregnancy complication*		
Yes	25	15.2
No	139	84.2
Missing	1	0.6

*Note*: Number of participants = 165. Ponderal index was calculated using the following formula: weight in kilograms divided by height in meters cubed. Height and weight were acquired during the neuroimaging visit. The participants kept indoor clothes on during the weighing. Distress score is a sum of Edinburgh Postnatal Depression Scale (EPDS) and Symptom Checklist 90 (SCL‐90) scores from that age. Maternal and paternal education data were combined from questionnaire data from 14 weeks gestation or 5 years of child age by choosing the highest degree reported. The three classes are: Low = Upper secondary school or vocational school or lower, Middle = University of applied sciences, High = University. On the question about alcohol usage, four subjects answered that they did not use alcohol during pregnancy, but also answered that they stopped using alcohol when they learned about the pregnancy. These were classified as “yes, stopped when learning about pregnancy”. The data for maternal monthly income estimate, alcohol use, and illicit drug use are from questionnaires at gestational week 14. The pregnancy complications include a diagnosis (according to ICD‐10) for O12 (Gestational edema and proteinuria without hypertension), O13 (Gestational hypertension without significant proteinuria), O14 (Severe pre‐eclampsia), O24 (Diabetes mellitus in pregnancy, childbirth, and the puerperium), O46 (Antepartum hemorrhage, not elsewhere classified), or O99.0 (Anemia complicating pregnancy, childbirth and the puerperium). Sex, birth weight, maternal BMI before pregnancy, and smoking data (combined with questionnaire data) were retrieved from the National Institute for Health and Welfare (www.thl.fi).

Abbreviations: BMI, body mass index, GW, gestational week, SD, standard deviation.

### 2.2 Bias assessment

Mothers of the children who did not participate in the neuropsychological visits (out of the 1288 contacted families) had a lower education level (χ^2^(2) = 30.94, *p* < .001), a lower monthly income (χ^2^(3) = 11.65, *p* = .009) and were younger (*t* (1286) = −4.130, *p* < .001) compared to the mothers in the families that participated in the neuropsychological visits.

Mothers of the children who participated in the neuropsychological visits but not in the neuroimaging visits were older (*t* (369) = 1.97, *p* = .047) but did not differ in education level or monthly income compared to the mothers in the families that participated in the MRI visit.

The children who participated in the neuropsychological visits but not in the MRI visits did not differ in PIQ, Block Design, or Matrix Reasoning performance from those that participated in the MRI visit.

### 2.3 Procedures

Non‐verbal ability was assessed at 5 years of age using the Block Design and Matrix Reasoning subtasks of the Wechsler Preschool and Primary Scale of Intelligence‐Third Edition (WPPSI‐III, Wechsler, 1967).

After the FinnBrain Child Development and Parental Functioning Lab visit, the participating families were invited to the MRI visit, where structural T1‐weighted images were collected as a part of max. 60‐min scan.

### 2.4 Neuropsychological study visits

The neuropsychological study visits for 5‐year‐old children included neurocognitive testing, eye‐movement tracking, mother–child interaction assessment, and questionnaires filled out by the parents. Neurocognitive testing included assessments of the child's general cognitive ability (WPPSI‐III subtests Block Design, Matrix Reasoning and Similarities) and executive functioning and self‐regulation, of which only the non‐verbal tasks from the general cognitive ability assessments are used in the current study.

The approximately two‐hour‐long study visits were conducted and video recorded by graduate students in quiet examination rooms and the data collection was overseen by PhD students/psychologists. The graduate students were trained by PhD students/psychologists prior to data collection, to ensure unified test administration among all students, and to ensure that the students had sufficient interaction skills to scaffold the children's motivation and mood during the study visit. Written informed consent was provided by the parents prior to the study visit, and the parents received feedback of the child's performance on some of the assessment methods after the study visit.

### 2.5 Non‐verbal ability

Non‐verbal ability was assessed using the Finnish version of WPPSI‐III, which is a standardized and widely used measure of cognitive ability in young children from ages 2 years and 6 months to 7 years and 3 months (Wechsler, 2009). In this study, a composite sum score of non‐verbal ability (PIQ; mean 100) was estimated using two subtests: the Block Design task measuring visuospatial ability and the Matrix Reasoning task measuring visual abstract reasoning. The standardized scale scores corresponding the raw scores of the subtests were based on Finnish norms and result in a mean of 10, reflecting standardized mean performance in the population at each age. Additionally, analyses of the subtests were conducted separately to get further information on the possible subtest driving the findings.

The PIQ scores were: mean = 104.7, SD = 15.4, range 68–146. Block Design scores: mean = 10.5, SD = 3.3, range 3–19. Matrix Reasoning scores: mean = 10.8, SD = 2.8, range 1–18. These results suggest a normally distributed cognitive ability in the sample of the present study. The Pearson correlation between PIQ and Block Design was 0.809 (*p* < .001), between PIQ and Matrix Reasoning was 0.711 (*p* < .001), and between Block Design and Matrix Reasoning was 0.164 (*p* = .039). Scatter plots of the cognitive measurements are shown in Figure S1.

### 2.6 Neuroimaging study visits

All visits were performed by research staff for research purposes. The participants were recruited via phone calls by the research staff. A staff member made a home visit to deliver practice materials, give further information about the study visit, and to answer any remaining questions. At the start of the study visit, written informed consent from both parents as well as verbal assent from the child were acquired. The visits had a two‐hour preparation time before the scan, which consisted of familiarization with the research staff, practice for the scan, and a light meal. The preparation time was long enough so that it allowed the staff to attend to the needs of the child. Participants were scanned awake or during natural sleep. A parent and a research staff member were present in the scanning room throughout the scan. Everyone in the room had their hearing protected with earplugs and headphones. During the scan, participants were allowed to watch a movie or a cartoon of their choice, apart from the functional MRI (fMRI) sequence. The study visit protocol has been described in more detail in our earlier work (Copeland et al., 2021; Pulli et al., 2022).

All images were viewed by one neuroradiologist (R.P.) who then consulted a pediatric neurologist (T.L.) when necessary. The protocol with incidental findings has been described in our earlier work (Kumpulainen et al., 2020). In the whole neuroimaging sample (*n* = 203), there were 13 participants with incidental findings (6.4%). Of them, 11 were included in the sample of this study (*n* = 165). Among the 11 incidental findings in this study, there were eight cerebellar anomalies (typically Chiari malformation), one vascular anomaly, and two pineal cysts. None of these affected the cortical analyses in this study.

### 2.7 MRI data acquisition

Participants were scanned using a Siemens Magnetom Skyra fit 3T with a 20‐element head/neck matrix coil. We used Generalized Autocalibrating Partially Parallel Acquisition (GRAPPA) technique to accelerate image acquisition (parallel acquisition technique [PAT] factor of 2 was used). The scanning protocol (maximum length 60 min) included a high‐resolution T1‐weighted Magnetization Prepared RApid Gradient Echo (=MPRAGE), a T2‐weighted Turbo Spin Echo (=TSE), a 7‐min resting state functional MRI, and a 96‐direction single shell (b = 1000 s/mm^2^) Diffusion Tensor Imaging (=DTI) sequence (Merisaari et al., 2019; Rosberg et al., 2022) as well as a 31‐direction with b = 650 s/mm^2^ and a 80‐direction with b = 2000 s/mm^2^. For the purposes of the current study, we acquired high resolution T1‐weighted images with the following sequence parameters: repetition time = 1900 ms, echo time = 3.26 ms, inversion time = 900 ms, flip angle = 9 degrees, voxel size = 1.0 × 1.0 × 1.0 mm^3^, field‐of‐view 256 × 256 mm^2^. The scans were planned as per recommendations of the FreeSurfer developers (https://surfer.nmr.mgh.harvard.edu/fswiki/FreeSurferWiki?action=AttachFile&do=get&target=FreeSurfer_Suggested_Morphometry_Protocols.pdf, at the time of writing).

### 2.8 Image processing

The cortical reconstruction and volumetric segmentation for all 165 images were performed with the FreeSurfer software suite, version 6.0.0 (http://surfer.nmr.mgh.harvard.edu/). We selected the T1 image with the least motion artefact (in case there were several attempts due to visible motion during scan) and then applied the “recon‐all” processing stream with default parameters. It begins with transformation to Talairach space, intensity inhomogeneity correction, bias field correction (Sled et al., 1998), and skull‐stripping (Ségonne et al., 2004). Thereafter, white matter is separated from gray matter and other tissues and the volume within the created gray–white matter boundary is filled. After this, the surface is tessellated and smoothed. After these preprocessing steps are completed, the surface is inflated (Fischl, Sereno, & Dale, 1999) and registered to a spherical atlas. This method adapts to the folding pattern of each individual brain, utilizing consistent folding patterns such as the central sulcus and the sylvian fissure as landmarks, allowing for high localization accuracy (Fischl, Sereno, Tootell, & Dale, 1999). FreeSurfer uses probabilistic approach based on Markov random fields for automated labeling of brain regions. Cortical thickness is calculated as the average distance between the gray–white matter boundary and the pial surface on the tessellated surface (Fischl & Dale, 2000). The cortical thickness measurement technique has been validated against manual measurements from imaging data (Kuperberg et al., 2003; Salat, 2004) and against postmortem histological analysis (Rosas et al., 2002).

After the initial FreeSurfer processing, we visually inspected the images for segmentation errors and manually edited all images. Briefly, the manual edits included removing skull fragments where they affected the pial border, correcting errors in the border between gray and white matter, and removing arteries. After the edits, the FreeSurfer recon‐all was run again. For a more detailed description of the image processing procedure, please see our previous article (Pulli et al., 2022).

### 2.9 Confounders

Based on previous studies and our own previous work on this age group (Silver et al., 2022), child sex, age at scan, ponderal index (mass in kilograms divided by height in meters cubed; measured during the neuroimaging visit), as well as maternal age at term and maternal education level were included as covariates in our analyses. Maternal education data were combined from questionnaire data from 14 weeks gestation or 5 years of child age by choosing the highest degree reported (three classes: Low = Upper secondary school or vocational school or lower, Middle = University of applied sciences, High = University; low and middle level education grouped together for statistical analyses). Ponderal index reflects the body size of the child, correlating with brain volume, similarly to sex in which case boys have larger brains on average. In child populations, age is relevant for cognitive ability as children are constantly developing as they age, and age at scan is also one of the most important predictors of brain metrics in developmental populations (Jha, Xia, Ahn, et al., 2018; Knickmeyer et al., 2016). Age at neuropsychological measurement significantly correlated with age at scan (Pearson *r* = .32, *p* < .0001) and we decided to use age at MRI scan as the age variable of interest. Maternal age at term covaries with both cognitive ability (Lyall et al., 2020) and brain structure (Shaw et al., 2012). Similarly, maternal educational level (and socioeconomic status more generally) is associated with both cognitive ability (González et al., 2020; Kenyhercz & Nagy, 2021; Wong & Edwards, 2013).

Additionally, some factors that could potentially affect the results, as they all have been shown to covary with both cognitive ability and brain metrics, were explored in sensitivity analyses: maternal pre‐pregnancy body mass index (BMI; Edlow, 2017; Li et al., 2016; Na et al., 2021; Ou et al., 2015; Shapiro et al., 2020), alcohol exposure in utero (mothers who continued to use alcohol after they learned about the pregnancy excluded; Archibald et al., 2001; Chasnoff et al., 2015; Donald et al., 2015; Nardelli et al., 2011), tobacco exposure in utero (mothers with any tobacco use during pregnancy excluded; Chang et al., 2016; El Marroun et al., 2014; Fried et al., 2003; Knickmeyer et al., 2016), preterm birth (participants born before GW 37 excluded; Aylward, 2014; Brydges et al., 2018; Jeong et al., 2016; Jha, Xia, Ahn, et al., 2018; Kapellou et al., 2006; Knickmeyer et al., 2016), prenatal distress (a sum of depressive and anxiety symptoms measured with the Finnish versions of the Edinburgh Postnatal Depression Scale (EPDS; Cox et al., 1987) and the Symptom Checklist‐90‐Revised (SCL‐90; Derogatis, 1994), respectively, from GW 14, 24, and 34)(Davis et al., 2020; Laplante et al., 2004), postnatal distress (a sum of depressive and anxiety symptoms measured with EPDS and SCL‐90, respectively, at child ages 3 and 6 months)(Koutra et al., 2013; Lebel et al., 2016; Sharp et al., 1995; Zou et al., 2019), and paternal education level (classified the same way as maternal education; González et al., 2020; Jha, Xia, Ahn, et al., 2018; Knickmeyer et al., 2016). Additionally, three different early life markers for potentially abnormal development were explored: 5 min Apgar score (Aoki et al., 2020; Hong & Lee, 2018), pregnancy complications (mothers with any complications excluded; Koparkar et al., 2021; Tuovinen et al., 2014; Xuan et al., 2020; Zheng et al., 2022), and stay in the neonatal intensive care unit (NICU; those with NICU stay excluded; Aoki et al., 2020).

There were some missing data. Eight participants were missing data for the alcohol exposure, and one was missing data for pregnancy complication and in these cases, we used mode imputation. One participant was missing pre‐pregnancy maternal BMI, and maternal distress scores were missing as follows: GW 14 = 6, GW 24 = 5, GW 34 = 4, 3 months = 13, and 6 months = 27. In these cases, we used mean imputation based on the 173 participants with usable structural MRI data. At each individual EPDS and SCL‐90 questionnaire, a maximum of 3 missing answers were allowed (otherwise they were labeled as missing) and the missing ones were imputed using the mean of other answers from that questionnaire. Finally, paternal education was only available for 108 participants. This data were not imputed, but instead the sensitivity analysis for paternal education level was done using only the participants with available data.

### 2.10 Statistics

Statistical analyses concerning confounders, demographics, and regions of interest (ROI) were conducted using the IBM SPSS Statistics for Windows, version 27.0 (IBM Corp., Armonk, NY, USA). Scatter plots and the related statistics were created using JASP version 0.16.1.0 (JASP Team, 2022). Correction for multiple comparisons was done using RStudio (version 2022.07.1, build 554; RStudio Team, 2020) command “p.adjust.” Statistical significance in all analyses was calculated two‐tailed at alpha level 0.05.

The associations between the covariates included in the main statistical model and regional brain metrics were estimated (for the full list of regions, see “Region of interest‐based analyses” below), the zero‐order correlations were calculated for continuous variables (Pearson correlations for maternal age at term and ponderal index, Spearman correlations for age at scan (as age at scan was not normally distributed)) and independent samples t‐tests for sex and maternal education level. We report raw *p*‐values in Table S1.

To assess potential selection bias, comparisons between the neuroimaging participants that were included in the final sample (*n* = 165) and those that were excluded (*n* = 38) were performed with independent samples t‐tests for continuous background factors and chi‐square tests for categorical factors. All background factors from Table 1 were examined. Information regarding alcohol exposure was missing for 12/203 participants and was analyzed with three different approaches: (1) missing = no exposure, (2) missing = exposure, and (3) missing data not imputed. For the other background factors, missing data were not imputed for this analysis.

#### 2.10.1 Vertex‐wise statistical analyses

For the purposes this study, we pre‐smoothed fsaverage surfaces as instructed by FreeSurfer manual for analyses with Query, Design, Estimate, Contrast (Qdec), a single‐binary application included in the FreeSurfer software suite (www.surfer.nmr.mgh.harvard.edu). Qdec is a graphical user interface for a statistics engine running a vertex‐by‐vertex general linear model (GLM). For display purposes, we used the standard FreeSurfer's fsaverage in MNI305 space (MNI = Montreal Neurological Institute). We tested for clusters with statistically significant associations between non‐verbal ability and cortical GM volume, surface area, and cortical thickness. The data were smoothed with a kernel of 10 mm full width at half maximum. A Monte Carlo Null‐Z Simulation was run with a z‐value threshold of 1.3, corresponding to *p* = .05 (Hagler et al., 2006). After the simulation, a z‐value threshold 1.3 was used for statistically significant clusters. For confounding factors and performed sensitivity analyses, please see “Confounders.” Age at scan was squared for the purposes of running Qdec. In the sensitivity analyses, we added the potential confounders to the model one at a time (continuous factors) or excluded the exposed group from the analysis (categorical factors). The one exception to this was the paternal education level, where the analysis was ran using only the participants with available data and paternal education replaced maternal education in the model.

#### 2.10.2 Region of interest‐based analyses

Additionally, we calculated partial correlations (controlling for participant sex, maternal education level, maternal age at term, participant ponderal index at scan, and participant age at scan) between (1) all cognitive measurements (PIQ, Block Design, and Matrix Reasoning), and (2) a multitude of brain metrics, including volume, surface area, and cortical thickness in all 68 ROIs in the Desikan–Killiany atlas (Desikan et al., 2006) as well as total surface area (separately for both hemispheres), mean cortical thickness (separately for both hemispheres), brain volume (excluding ventricles), and estimated total intracranial volume; in total 210 brain metrics per cognitive measurement. We excluded poor quality ROIs from this analysis (described in detail in our previous article Pulli et al., 2022). For this part of the analysis, we corrected for multiple comparisons using the Benjamini–Hochberg procedure (Benjamini & Hochberg, 1995) across all 630 comparisons. Finally, moderation effects of non‐verbal ability on the association between age and mean cortical thickness in the left and right hemispheres were estimated.

## 3 RESULTS

### 3.1 Demographics

The children from the neuroimaging sample (*n* = 203) that were included in this study (*n* = 165) had higher gestational age at birth (included 39.79 weeks, SD 1.57; excluded 39.01 weeks, SD 2.15; *p* = .041) and fewer had a NICU stay (included 142 no, 23 yes (13.9%); excluded 25 no, 11 yes (30.6%); χ^2^(1) = 0.016), and their mothers had lower pre‐pregnancy BMI (included 24.20, SD 4.33; excluded 25.83, SD 4.77; *p* = .044), compared to those who were excluded (*n* = 38).

### 3.2 Cortical gray matter volume and non‐verbal ability

**FIGURE 1:**
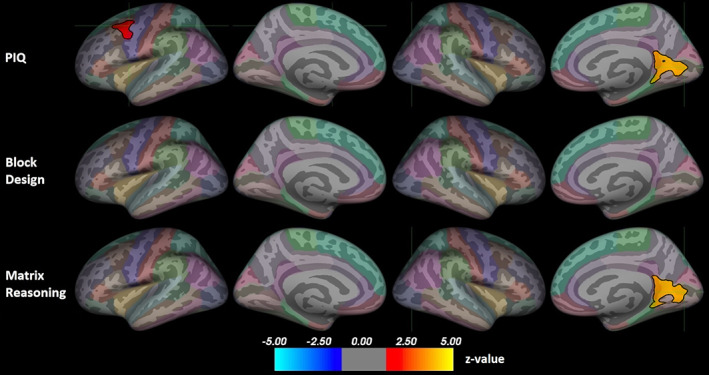
Positive associations between non‐verbal ability and cortical gray matter volume. Results corrected for multiple comparisons using Monte Carlo Null‐Z simulation. Color indicates significance as a z‐value. The position of the green crosshair indicates the most statistically significant vertex in statistically significant clusters. Left hemisphere on the left and right on the right side. Color coding of regions according to the Desikan–Killiany atlas. PIQ, performance intelligence quotient.

Figure 1 presents the associations between cortical GM volume and non‐verbal ability. All significant associations were positive. For PIQ, there were significant clusters in the left caudal middle frontal gyrus (peak z = 1.67, size = 950.9 mm^2^, peak coordinates −41.4, 3.5, 46.8) and the right pericalcarine region (peak z = 4.00, size = 1639.8 mm^2^, peak coordinates 14.3, −77.4, 4.8). There were no significant correlations between Block Design scores and brain volumes. For Matrix Reasoning, there was a significant cluster in the right pericalcarine region (peak z = 4.00, size = 1859.4 mm^2^, peak coordinates 14.3, −77.4, 4.8).

### 3.3 Pial surface area and non‐verbal ability

**FIGURE 2:**
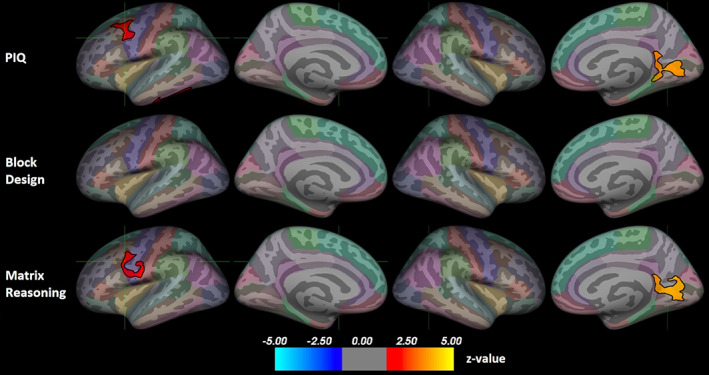
Positive associations between non‐verbal ability and pial surface area. Results corrected for multiple comparisons using Monte Carlo Null‐Z simulation. Color indicates significance as a z‐value. The position of the green crosshair indicates the most statistically significant vertex in statistically significant clusters. Left hemisphere on the left and right on the right side. Color coding of regions according to the Desikan–Killiany atlas. PIQ, performance intelligence quotient.

Figure 2 presents the associations between pial surface area and non‐verbal ability. All significant associations were positive. For PIQ, there were significant clusters in the left caudal middle frontal gyrus (peak z = 2.05, size = 870.7 mm^2^, peak coordinates −36.1, 0.6, 31.0), the left inferior temporal gyrus (peak z = 1.37, size = 692.4 mm^2^, peak coordinates −45.0, −18.5, −29.5), and the right lingual gyrus (peak z = 3.70, size = 1239.1 mm^2^, peak coordinates 25.4, −61.6, 0.7). There were no significant correlations between Block Design scores and surface area. For Matrix Reasoning, there were significant clusters in the left caudal middle frontal gyrus (peak z = 2.07, size = 874.9 mm^2^, peak coordinates −36.1, 0.6, 31.0) and the right lingual gyrus (peak z = 4.00, size = 1311.8 mm^2^, peak coordinates 25.4, −61.6, 0.7).

### 3.4 Cortical thickness and non‐verbal ability

**FIGURE 3:**
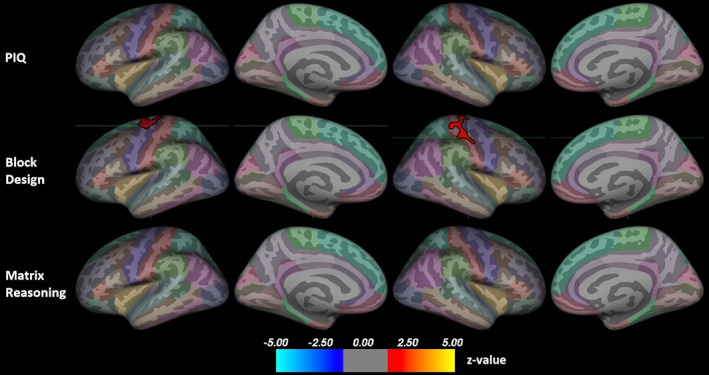
Positive associations between non‐verbal ability and cortical thickness. Results corrected for multiple comparisons using Monte Carlo Null‐Z simulation. The position of the green crosshair indicates the most statistically significant vertex in statistically significant clusters. Left hemisphere on the left and right on the right side. Color coding of regions according to the Desikan–Killiany atlas. PIQ, performance intelligence quotient.

Figure 3 presents the associations between cortical thickness and non‐verbal ability. All significant associations were positive. There were no significant correlations between PIQ and cortical thickness. For Block Design only, there were significant clusters in the left precentral gyrus (peak z = 1.70, size = 959.0 mm^2^, peak coordinates −36.8, −18.3, 64.5) and the right postcentral gyrus (peak z = 2.26, size = 1158.2 mm^2^, peak coordinates 48.8, −16.5, 49.1). There were no significant correlations between Matrix Reasoning scores and cortical thickness.

### 3.5 Sensitivity analyses

The correlations between PIQ and brain metrics are shown in Figures S2–S7. Qdec images are presented without correction for multiple comparisons at z‐value threshold 1.3. Overall, there are no large differences compared to the basic statistical model.

In the paternal education level model, there was a positive association between PIQ and cortical thickness in the left superior frontal cortex (peak z = 3.29, size = 1028.1 mm^2^, peak coordinates −7.1, 37.9, 25.1) that survived correction for multiple comparisons using Monte Carlo Null‐Z simulation (Figure S8). The cluster disappears if paternal education level is removed from the model or replaced with maternal education level. There were no associations between PIQ and either volume or surface area that survived correction for multiple comparisons.

### 3.6 ROI‐based analyses

There were no correlations between non‐verbal ability and either cortical volume, area, or thickness that survived correction for multiple comparisons (all adjusted *p*‐values ≥ 0.35). All raw *p*‐values are presented in Table S2.

### 3.7 Moderating effects of non‐verbal ability on the association between age and cortical thickness

The zero‐order Pearson correlations between age at scan and mean cortical thickness were *r* = −.056, *p* = .478 for the left hemisphere and *r* = −.030, *p* = .697 for the right hemisphere. The moderating effect of PIQ was not significant (*p* = .43 for the left and *p* = .81 for the right hemisphere).

### 3.8 Post hoc analyses

We further visually explored the regions where PIQ associated with brain metrics to assess whether either subtest was driving the results. Figure S9 presents the left caudal middle frontal gyrus where PIQ associated with both volume and surface area, as well as the left inferior temporal gyrus where PIQ associated with surface area. For the left caudal middle frontal gyrus, both subtests show some positive clusters in the same region. For the left inferior temporal gyrus, neither subtest alone shows large clusters in the region. In all three clusters, the results do not seem to be strongly driven by either subtest.

Figure S10 presents the right medial occipital region where PIQ associated with both volume and surface area. The Matrix Reasoning subtest shows large clusters in the same region for both volume and surface area, while the Block Design subtest shows barely any small clusters in the region, suggesting that the correlations between PIQ and brain metrics in the right medial occipital region were driven by the Matrix Reasoning task performance.

## 4 DISCUSSION

In this study, we examined the associations between non‐verbal ability and cortical brain structure (volume, surface area, and cortical thickness) in a sample of typically developing 5‐year‐olds. We hypothesized based on the P‐FIT model that non‐verbal ability would be positively correlated with volume and surface area in frontal and parietal regions. In line with the hypothesis, we found that the volume and surface area of the left caudal middle frontal gyrus were positively associated with non‐verbal ability. Additionally, we found significant positive associations with right medial occipital structure and left inferior temporal surface area. Furthermore, we expected to find associations between non‐verbal ability and cortical thickness in frontal and parietal regions. Two significant positive associations with visuospatial ability measures utilizing only one task were found but there were no associations with the overall non‐verbal ability of the child. Altogether, this is the first study to examine the cortical structural correlates of non‐verbal ability in a large sample of typically developing 5‐year‐olds. Our results suggest that some of the structures identified in studies of older participants are correlated with non‐verbal ability at this stage of development.

We found associations between non‐verbal ability and both volume and surface area in the caudal middle frontal gyrus. The middle frontal gyrus is a region that is often associated with cognitive ability both structurally (Basten et al., 2015; Botdorf & Riggins, 2018; Brouwer et al., 2014; Frangou et al., 2004; Schilling et al., 2013) and functionally (Basten et al., 2015; Osaka et al., 2004), however the findings are typically seen in the frontal parts of the middle frontal gyrus. Furthermore, the pediatric studies on the topic have typically focused on cortical thickness rather than volume or surface area (Botdorf & Riggins, 2018; Brouwer et al., 2014). The posterior parts of the caudal middle frontal gyrus form a part of the premotor cortex, which is, in addition to cognitive ability (Jung & Haier, 2007; O'Boyle et al., 2005), also a relevant region for mathematical ability (Navas‐Sánchez et al., 2016), working memory (an fMRI study, Osaka et al., 2004), and speech perception (based on transcranial magnetic stimulation studies, see Meister et al., 2007; Sato et al., 2009). The premotor cortex is especially often observed relevant in functional brain studies focusing on cognitive ability (Jung & Haier, 2007; Osaka et al., 2004), while structural findings are comparatively scarce. Navas‐Sánchez et al. (2016), observed larger surface area in math gifted adolescents compared to high cognitive ability controls especially in the left caudal middle frontal gyrus. Some of the tests used in the study by Navas‐Sánchez et al. (2016) were similar to ours, assessing visuospatial thinking and the ability to recognize patterns or rules, but they also measured other aspects of“mathematical giftedness,” such as intuition and creativity that may have completely different neurobiological correlates. Nevertheless, our results support the previous studies in proposing that the positive association between non‐verbal ability and surface area may already be observable at 5 years of age, which is in line with the finding that more intelligent children reach peak surface area faster (Schnack et al., 2015).

Volume and surface area in the right medial occipital region, including parts of the pericalcarine, isthmus of cingulate gyrus, precuneus, and lingual regions, were associated with non‐verbal ability, and more specifically with visual abstract reasoning rather than visuospatial ability. Some studies in adults have found associations between general cognitive ability and the lingual gyrus volume (Colom et al., 2006b) as well as more widespread occipital GM volumes (Colom et al., 2006a; Haier et al., 2004). Notably, Colom et al. only found associations with visuospatial ability (visual abstract reasoning ability not tested, Colom et al., 2006a), while our results in the occipital lobe were driven by visual abstract reasoning ability. Furthermore, in previous articles, the associations between general cognitive ability and occipital brain metrics have generally been found on the lateral rather than medial surface in adults (Colom et al., 2006a) and children/adolescents (Karama et al., 2009). One study in children has found cortical thickening in left medial occipital cortex to be associated with higher visuospatial ability (Sowell et al., 2004). Parts of the occipital lobe are often involved in functional studies on cognitive function (Jung & Haier, 2007). For example, bilateral inferior occipital gyri activation is seen during deduction tasks (Goel & Dolan, 2004). Bilateral precuneus shows increased activation during non‐verbal tasks in adolescents with high cognitive ability (Lee et al., 2006; O'Boyle et al., 2005). Notably, the activation is typically seen in more superior parts of the precuneus. To the best of our knowledge, this is the first study to link right medial occipital cortex volume and surface area to non‐verbal ability in children and thus, these areas should be included among the hypothesized structures related to non‐verbal ability specifically.

In both volume and surface area, we observed associations with visual abstract reasoning ability in largely similar areas than non‐verbal ability. On the contrary, there were no associations between volume or surface area and visuospatial ability. In contrast to our results, one previous study in adolescents measured both visual abstract reasoning and visuospatial ability (using the same subtests as we did) and found both to be associated with cortical thickness in the left frontal cortex, suggesting a common underlying neurobiology between visual abstract reasoning and visuospatial ability (Schilling et al., 2013). To further explore the possibility of a common neurobiological basis in our sample, we examined the Qdec analyses from the two subtests separately without correction for multiple comparisons. We observed a major difference between the clusters associated with visual abstract reasoning and visuospatial ability in the right medial occipital region, while the clusters in the prefrontal cortex were relatively similar. Our findings are not in conflict with previous findings that support the idea that the left middle frontal region is involved non‐verbal ability (Navas‐Sánchez et al., 2016; Schilling et al., 2013). On the other hand, our findings do suggest that the right medial occipital cortex volume and surface area are associated with visual abstract reasoning ability but not with visuospatial ability. However, to the best of our knowledge, this is the first study to find this connection and further studies are needed to confirm the findings.

We found a positive association between non‐verbal ability and surface area in the inferior temporal gyrus. The inferior temporal gyrus, as well as the medial occipital region, has a key role in the ventral visual pathway (Kravitz et al., 2013), a network responsible for object recognition. On a related note, it has a role in the visual and auditory word processing (Cohen et al., 2004). In children with a family risk for dyslexia, a smaller surface area was observed, even when controlling for their reading ability (Beelen et al., 2019). Additionally, one pediatric neuroimaging study found a positive association between general cognitive ability and inferior temporal cortical thickness in children (Karama et al., 2009). In theory, one would expect the structural and functional characteristics of the system responsible for object and pattern recognition to affect the performance on tasks that require pattern recognition (such as the tests in our study). Regarding the function of the ventral visual pathway, it has been hypothesized that the role of the central route (which includes the medial occipital and inferior temporal cortices) may be more important in early development compared to later life, when alternative routes in the network have been better established (Kravitz et al., 2013) and even damage to the central route in later life may only cause limited difficulties in recognition (Bertini et al., 2004). One study in young adolescents (Meruelo et al., 2019) found that cortical thickness in right medial occipital regions (including the posterior cingulate and the precuneus) and left temporal region (inferior temporal, as in our study, and additionally fusiform, a region relevant for face recognition) was positively associated with later academic achievement. These regions reflect our results well, but it is important to notice that both the brain and behavioral measurements were different. However, current information on the roles of temporal and occipital cortices for non‐verbal ability is conflicting (for review, please see Basten et al., 2015; Jung & Haier, 2007), and little is known about the role of these regions during childhood cognitive development.

We also found positive associations between cortical thickness and the visuospatial ability in the left precentral and right postcentral gyri. One previous study found widespread positive associations between Wechsler Abbreviated Scale of Intelligence (WASI) score and cortical thickness in 6–18‐year‐olds, also in older and younger halves separately (Karama et al., 2009). In their 6–12‐year‐old sample, the main overlap with our results is the positive association in the right postcentral gyrus. On the contrary, Botdorf and Riggins (2018) found no associations between general cognitive ability and cortical thickness in fronto–parietal regions but did find negative associations between cortical thickness and working memory (corrected for general cognitive ability) in multiple regions including the right postcentral gyrus in a sample of typically developing 4–8‐olds. The primary somatosensory area is not commonly associated with the cognitive ability in structural neuroimaging studies but when it is, the findings tend to be on the right rather than on the left hemisphere (Haier et al., 2004; Jung & Haier, 2007; Karama et al., 2009). Decreasing cortical thickness in the left precentral gyrus has been associated with a decrease in general cognitive ability in a sample where children and adolescents were imaged twice approximately 2 years apart (Burgaleta et al., 2014), suggesting that too much thinning during childhood development may be associated with undesirable outcomes.

The most recent studies suggest the cortical thickness peaks at a very young age, possibly even before 2 years of age (Bethlehem et al., 2022; Frangou et al., 2022). Therefore, in a simplistic “more advanced is better” interpretation, the participants with higher non‐verbal ability would be further in the developmental trajectory and have thinner cortices. Some studies have indeed found higher general cognitive ability (Schnack et al., 2015; Squeglia et al., 2013) and working memory (Botdorf & Riggins, 2018) to be associated with thinner cortex in children and adolescents. However, the positive associations seen in our study, while in agreement with many previous studies (Girault et al., 2020; Leonard et al., 2019; Meruelo et al., 2019; Schilling et al., 2013), contrast the idea that more advanced development would necessarily correlate with higher cognitive ability. One option to consider is that individuals may have different growth trajectories depending on cognitive ability. Shaw et al. (2006) have shown that the children with higher general cognitive ability reach their peak cortical thickness later, while Khundrakpam et al. (2017) suggest different cortical thickness coupling between the cortical regions between ages 6–18 years based on verbal ability. Meanwhile, other studies have found positive associations between general cognitive ability and cortical thickness in multiple brain regions in 6–18‐year‐olds (Karama et al., 2009; Karama et al., 2011), 9–24‐year‐olds (Menary et al., 2013), and adults (Bajaj et al., 2018) suggesting that individuals with higher general cognitive ability may retain a thicker cortex, although there have been conflicting results in adult studies, too (Tadayon et al., 2020). Overall, the results regarding cognitive ability and cortical thickness in children are currently inconsistent and more studies are needed. There are currently some large multisite neuroimaging projects devoted to longitudinal data collection of the developing brain, such as the HEALthy Brain and Child Development consortium (HBCD; Volkow et al., 2021) and the Adolescent Brain Cognitive Development consortium (ABCD; Hagler et al., 2019; Volkow et al., 2018), which will provide crucial information on developmental trajectories of the brain.

The cortical structural correlates of non‐verbal ability were similar for volume and surface area measurements but completely different for cortical thickness. This is in line with recent findings in both the similarity of volume and surface area metrics, and that they were more strongly associated with cognitive abilities than cortical thickness was (Michel et al., 2023). Volume is a combination of the two surface‐based measurements, cortical thickness and surface area, that reflect different biological features of the cortex. Specifically, cortical thickness is thought to reflect underlying biological processes including myelination (Natu et al., 2019), synaptic overproduction, and eventual pruning (Tierney & Nelson, 2009; Vidal‐Pineiro et al., 2020), while surface area reflects the number and spacing of cellular columns (Hill et al., 2010; Rakic, 1988). This is also reflected genetically, as both cortical thickness and surface area are highly heritable (0.81 and 0.89, respectively), but almost unrelated with each other (correlation 0.08; Panizzon et al., 2009) in adults, although the heritability of different brain metrics varies at different stages of development (Lenroot et al., 2009), and significantly higher correlations have been observed in the neonatal period (correlation 0.65; Jha, Xia, Schmitt, et al., 2018) and childhood/adolescence (correlation 0.63; J. E. Schmitt, Neale, et al., 2019). Although previous studies have found associations between cognitive ability and all three cortical brain metrics, one possible explanation for the discrepancy in our findings is a difference in effects sizes between brain metrics. A meta‐analysis including 37 studies of various age groups (McDaniel, 2005) found that total brain volume explained 11% of variation in cognitive ability (positive correlation). Regarding GM volume, specifically, Reiss et al. (1996) found that prefrontal GM volume predicted 20% of cognitive ability in 5–17‐year‐olds. In studies exploring cortical thickness and non‐verbal ability (Leonard et al., 2019; Schilling et al., 2013) or general cognitive ability (J. E. Schmitt, Raznahan, et al., 2019; Squeglia et al., 2013), effects this strong were not observed. Even in the study with the strongest correlations (J. E. Schmitt, Raznahan, et al., 2019), the effects were modest in young children and then increased toward early adolescence. One possible reason why cortical thickness findings were limited compared to volume and surface area results may be that the effects in the age were too small and would have required a bigger sample size to be observed.

A large proportion of the findings in our study were in the cortical regions that have already developed by 5 years of age, including the primary somatosensory region for cortical thickness and visuospatial ability (Bethlehem et al., 2022) and the primary visual cortex for both volume and surface area and non‐verbal ability (Hill et al., 2010). Regions that develop slower (such as frontal regions, see Bethlehem et al., 2022) might be affected by different growth trajectories between children of different levels of cognitive ability (Shaw et al., 2006). In the future, longitudinal studies from early childhood to adolescence (and adulthood) will be of great interest.

Left superior frontal region (and anterior cingulate gyrus) cortical thickness was positively associated non‐verbal ability but only when controlling for paternal education level. These regions are part of the P–FIT model (Frangou et al., 2004; Gong et al., 2005; Jung & Haier, 2007) and cortical thickness in the left frontal superior frontal region specifically (although on the lateral surface) was positively associated with non‐verbal ability in one previous study in adolescents (Schilling et al., 2013). Why this association was found only when corrected for paternal education is unclear, and in this study the smaller sample size for paternal education level analyses is a confounding issue. More studies are needed to confirm this finding.

Vertex‐wise and ROI‐based statistics were explored separately, and the ROI‐based analyses yielded no significant results. The significant clusters were relatively small compared to many Desikan–Killiany ROIs. Furthermore, many of the clusters appeared at the junctions of different ROIs. Therefore, effects would have to be large, for it to be reflected in the average value of the larger ROI. Based on our results, the structural correlates of non‐verbal ability are local and hence tentatively better captured by data‐driven clusters than predefined ROIs. Future studies could extend our findings by exploring functional differences associated with cognitive abilities.

Both a strength and a limitation of this study is the limited age range in a cross‐sectional setting. While the strength lies in the possibility to understand the neural correlates of non‐verbal ability at this specific age relevant for later development, it precludes true longitudinal and developmental interpretations. Especially with cortical thickness, it seems to be the case that longitudinal modeling is needed to find the potential individual differences in growth trajectories and how they might relate to non‐verbal ability. This was evident in the moderation analysis, where we did not find a correlation between age and cortical thickness, most likely because the age range was so limited that the trend of decreasing cortical thickness (Bethlehem et al., 2022) was not observed. Consequently, the lack of observed moderation effect may be due to the limited age range and cross‐sectional design. Longitudinal study designs are recommended to explore this question. On the other hand, to our knowledge, this is the first neuroimaging study to explore the association between non‐verbal ability and structural brain development in a large sample of typically developing 5‐year‐olds. The small age range provides an opportunity to get an accurate image of brain structure at this stage of development. Another limitation is the generalizability of the results especially to clinical samples. The participants in the final sample were born at a higher gestational age and had less visits to the NICU, suggesting that many participants with even slight issues during pregnancy or perinatal period were not included in the sample. Furthermore, our sample is highly ethnically homogenous, and the results are not necessarily generalizable to populations not of European descent.

## 5 CONCLUSION

To the best of our knowledge, this is the first study to explore cortical structural development in relation to non‐verbal ability in a large sample of typically developing 5‐year‐olds. We found that non‐verbal ability was associated with volume and surface area in left middle frontal and right medial occipital regions, and especially the medial occipital region was associated with visual abstract reasoning rather than visuospatial ability. On the other hand, cortical thickness in left precentral and right postcentral gyri were only associated with visuospatial ability specifically. Discrepancy between cortical thickness results and other results is not surprising considering that cortical thickness develops relatively independently from surface area and volume (Winkler et al., 2010), on a different trajectory (Bethlehem et al., 2022), and has a different genetic basis (espcially in adult research; Panizzon et al., 2009). Most associations between brain structure and non‐verbal ability were found in frontoparietal regions as expected based on the P‐FIT model (Jung & Haier, 2007), the most notable exception being the right medial occipital region. All associations were positive, which was also in line with previous literature in pediatric populations. Our findings in the right medial occipital region add to the literature by discovering a new region that should be considered in future studies exploring the mediating and moderating roles of cortical structure for cognitive development in young children. Overall, neural characteristics of cognitive development should be studied in samples of different ages and backgrounds. Longitudinal studies involving young children will be especially important to characterize the potential individual differences in developmental trajectories.

## AUTHOR CONTRIBUTIONS

**Elmo P. Pulli:** Conceptualization, formal analysis, investigation, visualization, writing—original draft. **Saara Nolvi:** Conceptualization, supervision, writing—original draft. **Eeva Eskola:** Writing—original draft. **Elisabeth Nordenswan, Eeva Holmberg, Anni Copeland, Venla Kumpulainen, Eero Silver, Ekaterina Saukko:** Investigation, writing—review & editing. **Harri Merisaari:** Methodology, software, writing—review & editing. **Jani Saunavaara:** Methodology, writing—review & editing. **Riitta Parkkola, Tuire Lähdesmäki, Eeva‐Leena Kataja, Riikka Korja:** Writing—review & editing. **Linnea Karlsson, Hasse Karlsson:** Conceptualization, funding acquisition, project administration, writing—review & editing. **Jetro J. Tuulari:** Conceptualization, funding acquisition, methodology, supervision, writing—original draft.

## CONFLICT OF INTEREST STATEMENT

The authors declare no conflicts of interest.

## Supporting information


Figures S1–S10.

Table S1.

Table S2.

## Data Availability

The Finnish law and ethical permissions do not allow open sharing of the data used in this study, but data access is possible via formal material transfer agreements (MTA). Investigators that wish to access the data are encouraged to contact Principal Investigator of the FinnBrain Birth Cohort study Hasse Karlsson (hasse.karlsson@utu.fi).
